# Editorial: Genetic Mutations Associated With Ocular Diseases

**DOI:** 10.3389/fcell.2021.815522

**Published:** 2021-12-24

**Authors:** Minzhong Yu, Rachida Bouhenni, Shree K. Kurup, Wei He

**Affiliations:** ^1^ Ophthalmic Research, Cole Eye Institute, Cleveland Clinic, Cleveland, OH, United States; ^2^ Department of Ophthalmology, Cleveland Clinic Lerner College of Medicine of Case Western Reserve University, Cleveland, OH, United States; ^3^ Department of Ophthalmology, University Hospitals, Case Western Reserve University, Cleveland, OH, United States; ^4^ Department of Pharmaceutical Sciences, Northeast Ohio Medical University, Rootstown Township, OH, United States; ^5^ The Vision Center, Akron Children’s Hospital, Akron, OH, United States; ^6^ Department of Ophthalmology, He Eye Specialist Hospital, He University, Shenyang, China

**Keywords:** eye, retina, cornea, glaucoma, myopia, genetic, mutation

Hereditary eye diseases affecting ocular cells and tissues are caused by mutations encoding many different signaling and structural proteins. The devastating effects of these diseases are substantial as they are often associated with several comorbidities leading to a poor quality of life. Many genetic mutations associated with ocular diseases such as the inherited retinal diseases (IRD) remain to be discovered. These molecularly unresolved IRDs are simplex/multiplex or autosomal recessive. In addition, the mechanisms of development of ocular disease for identified genetic mutations are not fully understood. This editorial summarizes the contribution of 16 original research papers, 1 brief research report and 1 review paper from some leading laboratories in this Research Topic. All of which has provided insight on important aspects of clinical and research related to ocular diseases caused by genetic mutations including glaucoma, cornea, nystagmus, myopia, and retina.

Primary Congenital Glaucoma (PCG) is caused by abnormal development of the trabecular meshwork and the anterior segment of the eye (Francois, 1980; Edward and Bouhenni, 2011; Li et al., 2011). PCG can be autosomal recessive or sporadic. CYP1B1, which is the most common causative gene in PCG harbors more than 140 distinct mutations that have been reported among various ethnic backgrounds (Li et al., 2011). Most patients with PCG caused by mutations in CYP1B1 have a severe disease phenotype. The protein belongs to the cytochrome p450 enzyme family and is involved in the metabolism of a variety of substrates, including steroids and retinoids that can act as morphogens during development (Choudhary et al., 2004) but its role in the eye is unknown Amirmokhtari et al. showed that absence of Cyp1b1 alone in a murine model is not sufficient to alter the visual function but does increase the retinal ganglion cells (RGC) susceptibility to axonopathy following pressure elevations. Thus, CYP1B1 may contribute to the ability of RGCs to respond to stress/injury through internal or external signaling mechanisms possibly mediated through bioactive metabolites.

Corneal dystrophies (CDs) are a group of genetic ocular diseases caused by abnormal substance accumulation in the cornea leading to a significant vision loss in some patients. Li et al. characterized the genetic landscape and mutation spectrum of patients with CDs in a large Han ethnic Chinese Cohort with IEDs and identified 2,334 distinct high-quality variants on 22 CD-related genes providing an important reference research baseline data for East Asia and other populations with DCs. Keratoconus is a type of CDs, that is challenging to be identified in early or subclinical stages. Chen et al. performed a mutational screening of VSX1, TGFBI, and ZEB1 genes and a full clinical assessment in 79 Chinese and 9 Greek families with keratoconus and found five variants in VSX1 and TGFBI genes that were identified in four families. Interestingly, very early corneal changes in corneal topography together with co-segregated variants were revealed in the relatives who were asymptomatic, suggesting that VSX1 and TGFBI genes may cause keratoconus through an autosomal dominant inheritance pattern with variable expressivity. Their study indicated that genetic testing is an important supplementary approach in re-classifying the disease manifestation and refining the pre-operative phase of refractive surgery.

Congenital nystagmus (CN) is a genetically and clinically heterogeneous ocular disorder that manifests as involuntary, periodic oscillations of the eye. Multiple modes of inheritance of CN have been reported, with the X-linked being the most common form. To date, only FRMD7 and GPR143 are considered the major disease-causing genes for CN. The FRMD7 gene was identified in both X-linked and sporadic CN about 15 years ago (Tarpey et al., 2006). Since then, more than 90 mutations in FRMD7 and 100 mutations in GPR143 have been reported. However, despite years of research, the pathogenesis of CN remains unclear. Results from Wang et al.’s study broadened the mutation spectrum of FRMD7 and GPR143, revealing significant differences in the screening rate between different groups of participants, thus providing new insights pursuing a precise diagnosis and genetic counseling for CN.

Using whole exome sequencing (WES), Liu et al. investigated genetic mutations in a cohort of 27 families with high myopia from Northwest China and found four new candidate genes, expanding the mutation spectrum and providing clues for further genetic study of high myopia. High myopia with alopecia areata in the occipital region has been reported in Knobloch syndrome and is caused by COL18A1 mutations. In the study of Wayng et al., other genetic causes of early onset high myopia (eoHM) in patients with alopecia areata in the cranial midline was studied. The scalp defects were found in patients with LAMA1 mutations. In six patients with eoHM and alopecia areata in the cranial midline, eight novel and one known loss-of-function mutations were detected. This included biallelic COL18A1 mutations in three patients with scalp leisure in the occipital region and biallelic LAMA1 mutations in the other three patients with scalp leisure in the parietal region. This study found that eoHM with midline alopecia areata could be associated with mutations in the LAMA1 in addition to COL18A1.

ARL3, a novel pathogenic gene of IRDs, encodes the ADP-ribosylation factor, (Arf)-like protein 3. Fu et al. identified two novel compound heterozygous pathogenic variants of ARL3. These variants could destabilize ARL3 protein and impair its interaction with RP2 protein. These findings suggested that the two compound heterozygous variants (c.91A>G, p.T31A; c.353G>T, p.C118F) were associated with early onset recessive cone-rod dystrophy (CRD), while c.91A>G might also be associated with a late onset of dominant CRD. This study extended both the genotype and phenotype of ARL3 associated IRD and highlighted the importance of protein stability and protein interactions in CRD pathogenesis. Using a multi-generation autosomal dominant family with progressive retinal degeneration and maculopathy, Ratnapriya et al. reported a novel, rare, heterozygous variant (p.Asp67Val) in ARL3 as the associated mutation. This soluble small GTPase has been localized to photoreceptor cilia suggesting that mutations in ARL3 can cause retinal ciliopathy providing new insights into the mechanisms of the disease which may lead to novel therapeutic options.

By investigating ABCA4 variants associated with IRDs, Zhu et al. reported 30 unique variants including four novel ones in a cohort with different retinopathies. Profiling of the ABCA4 mutations and their related clinical phenotypes will be helpful in understanding the phenotype/genotype correlation, mutation screening, prognostic counseling, and making decisions in selecting cases for gene therapy. In addition, Bohórque et al. studied 92 patients with IRD and identified 120 pathogenic or likely pathogenic variants, in which 30 of them were novel. In their CRD patients, ABCA4 was the most common mutated gene, while USH2A was predominant among the retinitis pigmentosa patients. Ten families carried pathogenic variants in more than one inherited retinal dystrophy gene. Two deep-intronic variants previously described as pathogenic in ABCA4 and CEP290 were identified.

Habibi et al. identified six potentially pathogenic variants in four genes in four families with autosomal dominant retinal dystrophies (RD) in recessive generations. A new digenetic combination was identified in an index patient with enhanced S-cone syndrome in one family: a heterozygous variant in RHO, and a homozygous pathogenic variant in NR2E3. Helicoid subretinal fibrosis associated with recessive NR2E3 variant p.[R311Q];[R311Q] was identified in another family. A new frameshift variant in RDH12 was identified in the third family with CRD. In the fourth family, the compound heterozygous variants p.[R964*];[W758*] were identified in IMPG2 associated with RP. Both affected parents and offspring were homozygous for the same variant in all families. These results suggest that the autosomal recessive trait of RD can be transmitted as pseudodominant inheritance in consanguineous families, extending the knowledge of pathogenic variants in RD genes.

Inactivating variants and a missense variant in the centrosomal CEP78 gene have been reported in autosomal recessive CRD with hearing loss (CRDHL); a rare syndromic inherited retinal disease which is distinct from Usher syndrome. A complex structural variant (SV) implicating CEP78 has been identified in CRDHL. Ascari et al. expanded the genetic architecture of typical CRDHL by the identification of complex SVs of the CEP78 region and characterization of their underlying mechanisms. Two novel canonical CEP78 splice variants and a frameshif single-nucleotide variant (SNV), two SVs affecting CEP78 were identified in three unrelated individuals with CRDHL. This study concluded that the CEP78 locus is prone to distinct SVs and that SV analysis is useful for genetic workup of CRDHL.

In retinitis pigmentosa (RP), the phenotype and genotype are heterogeneous making it challenging to investigate the genotype-phenotype correlation. EYS gene is one of the most prevalent causative genes of RP (Huang et al., 2015; Genet Med 2015). Xu et al. demonstrated the characteristics of RP genotypes and phenotypes by exploring an extensive global data from 420 IRD patients and 262 variants, including 19 novel mutations. This study expanded the genotypic landscape of EYS-associated IRD, and provided critical information for clinical genetic diagnosis and clinical consultation of EYS-associated IRD patients. Besides, RP1 truncation variants, including frameshift, nonsense, and splicing are common causes of RP. RP1 is a unique gene where truncations cause either autosomal dominant RP (adRP) or autosomal recessive RP (arRP) depending on the location of the variants. Wang et al. clarified the boundaries between adRP and arRP caused by RP1 truncation variants based on a systemic analysis of 165 RP1 variants from their in-house exome sequencing data of 7,092 individuals and a thorough review of 185 RP1 variants from the literature. The boundaries between dominant and recessive RP1 truncations were confirmed and refined. In addition, valuable questions for further investigations were suggested.

Central serous chorioretinopathy (CSC) is a common and heterogeneous chorioretinal disorder, while age-related macular degeneration is a disease characterized by a degenerative macula during aging. CSC and AMD have some similar clinical manifestations with CFH being the genetic risk locus for both suggesting some possible common pathophysiologic mechanisms. By comparing the genetic effects of 38 SNPs between CSC and AMD, Feng et al. revealed significant, complex genetic pleiotropic effects between the diseases.

Bardet-Biedl syndrome (BBS) is a rare genetic disease that affects multiple organs leading to a poor quality of life in late adolescence or early adulthood. In the study of Meng et al., the morphologic, functional and molecular characteristics of BBS were analyzed in 12 patients from 10 Chinese families. A total of five known and twelve novel variants in four BBS genes were identified. All patients displayed typical but heterogeneous retinal images of RP, unrecordable or severely damaged ffERG, mfERG, and PVEP. This study demonstrates the heterogeneity of the ocular characteristics of BBS. It also confirms the usefulness of a combination of the ffERG and FVEP assessments of visual function in the advanced stage of retinopathy in BBS.

Circular RNAs (circRNAs) are generated by a back-splicing mechanism with a covalently closed loop structure. Zhang et al. summarized the role and mechanisms of action of circRNAs in regulating gene expression and epigenetic modification, translating into peptides, and producing pseudogenes. The authors emphasized the relationship of circRNA research in ophthalmologic diseases. CircRNAs are promising biomarkers for diagnosis, assessment of progression and prognosis. Interventions targeting circRNAs may help develop new therapies for these diseases.

Overall, the 18 articles in this Research Topic provide insight on the molecular mechanisms of IRD and developmental eye diseases.
